# FDG-Avid Portal Vein Tumor Thrombosis from Hepatocellular Carcinoma in Contrast-Enhanced FDG PET/CT

**Published:** 2015

**Authors:** Xuan Canh Nguyen, Dinh Song Huy Nguyen, Van Tan Ngo, Simone Maurea

**Affiliations:** 1 Unit of PET/CT and Cyclotron, Choray Hospital, Vietnam; 2 Department of Liver Tumor, Choray Hospital, Vietnam; 3 Dipartimento Di Scienze Biomediche Avanzate, Facoltá Di Medicina E Chirurgia, Università Degli Studi Di Napoli Federico II, Italia

**Keywords:** FDG, Hepatocellular carcinoma (HCC), PET/CT, Portal vein tumor thrombosis (PVTT)

## Abstract

**Objective(s):**

In this study, we aimed to describe the characteristics of portal vein tumor thrombosis (PVTT), complicating hepatocellular carcinoma (HCC) in contrast-enhanced FDG PET/CT scan.

**Methods:**

In this retrospective study, 9 HCC patients with FDG-avid PVTT were diagnosed by contrast-enhanced fluorodeoxyglucose positron emission tomography/computed tomography (FDG PET/CT), which is a combination of dynamic liver CT scan, multiphase imaging, and whole-body PET scan. PET and CT DICOM images of patients were imported into the PET/CT imaging system for the re-analysis of contrast enhancement and FDG uptake in thrombus, the diameter of the involved portal vein, and characteristics of liver tumors and metastasis.

**Results:**

Two patients with previously untreated HCC and 7 cases with previously treated HCC had FDG-avid PVTT in contrast-enhanced FDG PET/CT scan. During the arterial phase of CT scan, portal vein thrombus showed contrast enhancement in 8 out of 9 patients (88.9%). PET scan showed an increased linear FDG uptake along the thrombosed portal vein in all patients. The mean greatest diameter of thrombosed portal veins was 1.8 ± 0.2 cm, which was significantly greater than that observed in normal portal veins (*P*<0.001). FDG uptake level in portal vein thrombus was significantly higher than that of blood pool in the reference normal portal vein (*P*=0.001). PVTT was caused by the direct extension of liver tumors. All patients had visible FDG-avid liver tumors in contrast-enhanced images. Five out of 9 patients (55.6%) had no extrahepatic metastasis, 3 cases (33.3%) had metastasis of regional lymph nodes, and 1 case (11.1%) presented with distant metastasis. The median estimated survival time of patients was 5 months.

**Conclusion:**

The intraluminal filling defect consistent with thrombous within the portal vein, expansion of the involved portal vein, contrast enhancement, and linear increased FDG uptake of the thrombus extended from liver tumor are findings of FDG-avid PVTT from HCC in contrast-enhanced FDG PET/CT.

## Introduction

In Vietnam, liver cancer is the second most common cancer after lung cancer in males and the fourth most common in females after breast, cervical, and stomach cancers. The Age Standardized Rate (ASR) of liver cancer is 25.3 in males and 5.9 in females per 100.000 in Ho Chi Minh City (1).

Hepatocellular carcinoma (HCC) is the most common type of liver cancer. Portal vein thrombosis (PVT) is a relatively common complication of HCC with an incidence rate of 31% (2). PVT is associated with poor prognosis and low survival rate (3-5); PVT also occurs in 4.5% of cases with liver cirrhosis (6). In addition, chronic hepatitis B virus is a major risk factor for the development of PVT (7).

The nature of PVT in HCC can be either malignant (due to tumor invasion) or benign (blood clots). Discrimination between malignant and benign PVT groups is important in patients’ prognosis and treatment selection. Although the biopsy of thrombus for histopathological examination is considered the gold standard for differentiating between malignant and benign PVT, multiple imaging modalities including contrast-enhanced ultrasound (CEUS) (8-11), contrast-enhanced computed tomography (CT) (12-14), and magnetic resonance imaging (MRI) (13, 15) have been used for this purpose.

Ultrasound is the initial imaging modality of choice for PVT investigation. Contrast enhancement of the thrombosed portal vein is the criterion for detecting this malignancy. The sensitivity of CEUS has been estimated at 88-100% for diagnosing malignant PVT (8-9). In fact, CEUS has been reported to be significantly superior to CT for the detection and characterization of PVT, complicating HCC (11). However, ultrasound contrast agents are not yet clinically available in some medical centers. The enhancement of PVT in the arterial phase and the expansion of involved portal veins are specific findings of malignant PVT in contrast-enhanced CT or MRI (12-15).

Positron emission tomography/computed tomography (PET/CT) with 2-deoxy-2-^18^F-fluoro-D-glucose (FDG) has been extensively used for the diagnosis, staging, treatment, and monitoring of different types of cancer.

Glucose metabolism in HCC tumors varies based on histological grades, showing high FDG uptake for poorly differentiated carcinomas and low FDG uptake for well differentiated ones (16). The sensitivity of FDG PET and PET/CT are relatively low in the diagnosis of HCC (16-17). However, FDG PET/CT has been reported to be valuable in the detection of recurrent HCC after treatment (18-19) and portal vein tumor thrombosis (PVTT) (20-23).

In this study, we performed FDG PET/CT along with dynamic contrast-enhanced CT for detection of suspected HCC recurrence as well as for the initial diagnosis of HCC in few other patients. The aim of this study was to describe the characteristics of FDG-avid PVTT from HCC in contrast-enhanced FDG PET/CT.

## Methods

This retrospective study included 9 HCC patients with FDG avid PVTT diagnosed by contrast-enhanced FDG PET/CT during May 2011-September 2013. Definition of FDG-avid PVTT was based on combination of portal venous phase contrast-enhanced CT and FDG PET findings. The criteria of PVTT consisted of (1) the thrombus identified as hypoattenuating intraluminal filling defect within the portal vein in the portal venous phase CT images, and (2) this thrombus appeared increased FDG uptake compared with the blood pool of reference normal portal vein and normal liver structures in FDG PET images. PET and CT DICOM images of patients were imported into the PET/CT imaging system for the re-analysis of contrast enhancement characteristics and FDG uptake in thrombus, the diameter of the involved portal vein, and characteristics of liver tumors and metastasis.

### Contrast-enhanced FDG PET/CT technique

All patients were fasted for at least 4 hours before performing contrast-enhanced FDG-PET/CT study. No one presented with renal failure or prior allergy-like reaction to the contrast media. Blood glucose level (finger-prick test) was measured to be 101.9±13.7 mg/dl (range: 78-128 mg/dl) before FDG administration. The patients were injected 5.18 MBq/kg (0.14 mCi/kg) of FDG. The scan was performed 60 minutes after FDG injection in a 64-slice PET/CT scanner (Biograph True D w/true V, Siemens Medical System).

A technique combining dynamic contrast-enhanced CT of the liver, multiphase imaging, and whole-body PET scan was performed. The CT scan sequence included non-contrast CT scan of the liver, hepatic arterial phase CT, portal venous whole-body CT, and equilibrium phase in contrast-enhanced CT scan of the liver. For the arterial phase, a contrast medium (300 mg of iodine per milliliter) of iopromide (Ultravist) or iopamidol (Iopamiro) was used with a dose of 1.2 ml per kg of body weight, infused at a rate of 3 ml per second, following 50 ml of normal saline chaser at a rate of 3 ml per second.

A threshold of 100 HU, set in the region of interest (ROI) at the lower part of descending thoracic aorta, triggered the start of hepatic arterial scan. The portal venous whole-body and equilibrium phases were performed approximately 65 and 120 seconds after beginning the infusion of contrast medium. During the portal venous phase, the patients were asked to breathe softly.

Afterwards, whole-body PET scan was performed as the field of whole body portal venous phase contrast-enhanced CT scan in a three-dimensional mode with an axial field view of 21.6 cm, a slice thickness of 5 mm, and axial and transaxial resolutions (FWHM @ 1cm) of 4.7 and 4.2 mm, respectively. The portal venous whole-body phase images were used for attenuation correction and fusion with PET images. The results of PET/CT were interpreted by a nuclear medicine physician and a radiologist.

### Analysis of contrast-enhanced FDG PET/CT

We analyzed the characteristics of thrombus in the arterial phase CT, the greatest diameter of thrombosed portal vein in comparison with the reference portal vein in the portal venous phase CT images, the FDG uptake of thrombus in comparison with the blood pool of remaining normal portal vein, and the location of thrombus in relation to liver tumors. The characteristics of liver tumors including contrast enhancement, number, the largest size, FDG uptake level, and extrahepatic metastasis were also analyzed.

FDG uptake is generally represented by standardized uptake value (SUV). SUV was calculated as radioactivity in volume-of-interest (Bq/ml)×body weight (kg)/injected radioactivity (Bq). Maximum SUV (SUV_max_) was the highest SUV value, measured in the study.

### Follow-up after FDG PET/CT studies

The type of therapy and survival time after FDG PET/CT evaluations were recorded for all patients.

## Results

### Patient characteristics

Nine patients (8 males and 1 female) with HCC and FDG-avid PVTT were included in this study. The mean age of the patients was 56.9±11.7 years (range: 38-76 years). HCC was diagnosed by histopathology in 4 patients and by a combination of clinical features, diagnostic imaging, and alphafetoprotein (AFP) in 5 patients. Four patients had

underlying cirrhosis and 4 patients had chronic viral hepatitis.

Prior history of alcohol consumption was not reported in patients. Two patients presented with de novo HCC. Seven patients with a previous history of HCC received specific treatments including radiofrequency ablation (RFA), transcatheter arterial chemoembolization (TACE), surgery, oral sorafenib, and Yttrium-90 microsphere. FDG PET/CT was performed during sorafenib therapy for 2 patients and approximately 3 months (range: 2-4 months) after other therapies. The mean AFP level was 6552±13516 ng/ml (range: 2-41163), measured almost 2 weeks before performing FDG PET/CT studies. The characteristics of patients are demonstrated in Table 1.

**Table 1:** Characteristics of 9 HCC patients of FDG-avid PVTT

Parameters		Value	
Mean age (yrs)		56.9 ± 11.7	(range: 38–76)
Gender	Male	8/9 pts	(88.9%)
	Female	1/9 pts	(11.1%)
Diagnosis of HCC by	histopathology	4/9 pts	(44.4%)
	clinical features, imaging, and AFP	5/9 pts	(55.6%)
	Cirrhosis	4/9 pts	(44.4%)
Viral hepatitis	B	2/9 pts	(22.2%)
	C	1/9 pts	(11.1%)
	B & C	1/9 pts	(11.1%)
Specific treatment	Yes	7/9 pts	(77.8%)
	No	2/9 pts	(22.2%)
AFP	>1000 (ng/mL)	4/9 pts	(44.4%)
	400-999 (ng/mL)	1/9 pts	(11.1%)
	<400 (ng/mL)	4/9 pts	(44.4%)
	Mean±SD (ng/mL)	6552±13516	(range: 2-41163)

*pts: patients*

### Characteristics of FDG-avid PVTT in contrast-enhanced FDG PET/CT images

**Figure 1:**
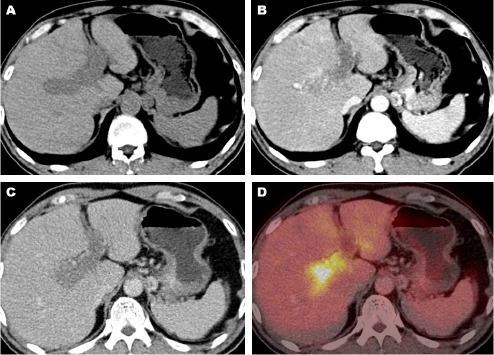
The contrast-enhanced FDG PET/CT image shows the expansion of right and left portal veins with hypodense intraluminal thrombus in non-enhanced CT scan (A). The thrombus in the right branch showed a linear contrast enhancement in the arterial phase CT (B). The thrombus appeared washout in the portal venous phase CT (C), and showed an increased FDG uptake in the PET/CT image (D)

**Figure 2:**
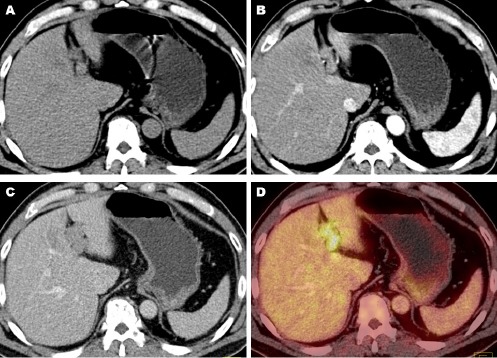
The contrast-enhanced FDG PET/CT shows the tumor thrombosis within the left expansile portal vein. The thrombus appeared isodense in the non-enhanced CT image (A), homogeneously contrast-enhanced in the arterial phase CT (B), and washed out in the portal venous phase CT (C). It showed an increased FDG uptake in the PET/CT image (D)

**Figure 3:**
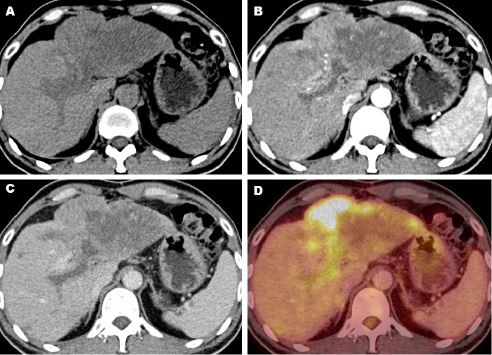
The contrast-enhanced FDG PET/CT shows a liver tumor with heterogeneous mild contrast enhancement and increased FDG uptake, adjacent to the left expansile thrombosed portal vein. The thrombus appeared hypodense in the non-enhanced CT image (A). It showed perithrombus contrast-enhancement in the arterial phase CT (B), washed out in the portal venous phase CT (C), and showed an increased FDG uptake in the PET/CT image (D)

**Figure 4:**
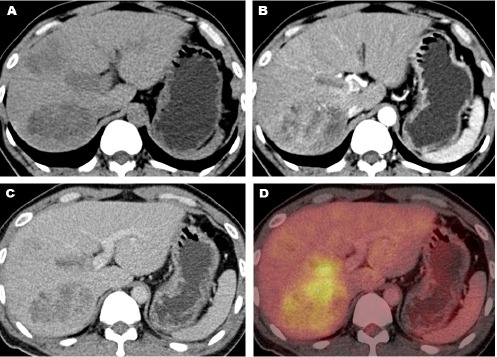
The contrast-enhanced FDG PET/CT shows a liver tumor with heterogeneous contrast enhancement and increased FDG uptake, adjacent to the right expansile thrombosed portal vein. The thrombus appeared hypodense in the non-enhanced CT (A). It showed perithrombus contrast enhancement in the arterial phase CT (B), appeared washout in the portal venous phase CT (C), and showed an increased FDG uptake in the PET/CT image (D)

PVTT involved the right branch of the portal vein in 3 out of 9 patients (33.3%), both branches in 2 patients (22.2%), the main portal vein and bilateral branches in 2 patients (22.2%), the main portal vein along with left branches in 1 patient (11.1%), (Figure 1-4). Eight patients (88.9%) had PVT appearing in the arterial phase contrast-enhanced CT. The contrast-enhanced patterns were irregular and linear in 5 patients (Figure 1) and perithrombus enhancement was observed in 3 patients (Figure 3-4).

PET scan showed an increased linear FDG uptake along the thrombosed portal veins in all patients (Figure 1-4). FDG uptake in the portal vein thrombi of second-order branches was identified in 6 out of 9 patients (66.7%). The characteristics of PVT, normal portal vein, and liver tumors in FDG PET/CT scan are shown in Table 2.

**Table 2:** Characteristics of thrombosed portal vein, reference normal portal vein, and liver tumors

Pt	Thrombosed portal vein			Reference normal portal vein			Liver tumors		
	Site	The greatest diameter (cm)	Thrombus SUV_max_	Site	The greatest diameter (cm)	Blood pool SUV_max_	Number	The greatest size (cm)	Tumor SUV_max_
1	R	1.7	9.3	L	1.4	2.3	3	14	11.8
2	R-L	1.6	4.8	M	1.4	1.8	2	13.5	11.4
3	L	1.8	8.1	R	1.6	2.6	>4	7.0	7.8
4	R-L-M	2.1	11.5	M*	1.7	2	1	12.0	12.1
5	L-M	1.7	6.8	R	1.4	3.4	1	7.2	8.6
6	R-L-M	2.2	5.4	M*	1.6	2.5	>4	2.5	4.5
7	R-L	1.7	5.2	M	1.4	2.6	1	3.5	6.4
8	R	1.9	7.4	L	1.5	2.3	2	8.7	8.6
9	R	1.5	4.5	L	1.3	2.5	1	6	3.8

Pt: patient, R: right portal vein, L: left portal vein, M: main portal vein, M

M*: the normal part of main portal vein

The mean greatest diameter of thrombosed portal vein was 1.8±0.2 cm, which was significantly greater than that of the normal portal vein (with a diameter of 1.5±0.2 cm) (P<0.001). The FDG uptake level in the thrombosed portal vein with SUV_max_ of 7.0±2.3 was significantly higher than that of the blood pool (SUV_max_ of 2.4±0.4) in the normal portal vein (*P*=0.001) (Table 3); a ratio of 3 (range: 1.8–5.8) was calculated on the FDG uptake of portal vein thrombus over the blood pool.

**Table 3:** The greatest diameter and FDG uptake in the thrombosed portal vein and reference normal portal vein

Parameters	Mean±SD	Range	*P*-value
Greatest diameter of the thrombosed portal vein (cm)	1.8 ± 0.2	1.5 - 2.2	<0.001
Greatest diameter of the reference normal portal vein (cm)	1.5 ± 0.2	1.3 - 1.7	
SUV_max_ of the thrombosed portal vein	7.0 ± 2.3	4.5 - 11.5	0.001
SUV_max_ of blood pool in the reference normal portal vein	2.4 ± 0.4	1.8 - 3.4	

All patients had visible liver tumors with contrast enhancement in the arterial phase CT images, contrast-washout in the portal venous and equilibrium phase CT, and increased FDG uptake in PET images (Figure 3-4). Four patients had 1 liver tumor, 2 patients had 2 liver tumors, 1 patient had 3 liver tumors, and 2 subjects had more than 4 liver tumors. The mean size of the greatest liver tumor was 8.3±4.2 cm (range: 2.5–14 cm). The mean liver tumor SUV_max_ was 8.3±3.1 (range: 3.8–12.1), which was not significantly higher than that of the thrombosed portal vein (*P*=0.12).

PVTT was identified by the direct extension of liver tumors in all patients. Eight out of 9 patients (88.9%) had adjacent liver tumors with direct invasion to the first- (6 patients) and second-order branches of portal veins (2 patients), visualized by dynamic CT and PET images. One remaining patient had an irregularly increased FDG uptake in multiple liver tumors and the surrounding liver tissues, extending to the contiguous portal veins. Five out of 9 patients (55.6%) had no extrahepatic metastasis, 3 patients (33.3%) had metastasis in only abdominal regional lymph nodes, and 1 subject (11.1%) had metastasis of abdominal regional lymph nodes, lung, bone, and brain.

After FDG PET/CT study, 7 out of 9 patients (77.8%), receiving supportive treatments, died during a mean follow-up time of 4.7 months (range: 1-9 months). Among 2 patients with oral sorafenib therapy (Nexavar), one remained alive after a 3-month follow-up, and 1 subject could not be contacted. The median survival time of patients was estimated at 5 months, based on Kaplan-Meier method.

## Discussion

The current study presented cases of FDG-avid PVTT from HCC patients diagnosed with contrast-enhanced FDG PET/CT scan. The characteristics of PVTT consisted of linear hypermetabolism, contrast enhancement of the thrombus, expansion of the involved portal veins, and extension of liver tumors. We selected cases of the portal vein thrombi with increased FDG uptake compared with blood pool of the reference normal portal vein and normal liver structures in contrast-enhanced FDG PET/CT, instead of comparison with the blood pool of mediastinum (23), or normal liver structures and/or the descending aorta (21, 24) in non-enhanced FDG PET/CT. Sharma P et al. investigated 24 patients with a known malignancy, accompanying FDG-avid venous thrombosis, and reported that both malignant and benign venous thrombi were hypermetabolic; malignant venous thrombi showed a more significant FDG uptake, compared to benign venous thrombi. Although in the mentioned study, only 5 HCC cases were reported (among 24 patients with various types of cancer). All hypermetabolic PVT in HCC was confirmed to be malignant in 100% of cases (23).

Sun L et al. analyzed FDG PET/CT images of 7 cases with HCC, complicated by PVT, and reported an increased FDG uptake of thrombi in all 5 cases of malignant PVT, while benign PVT did not appear FDG-avid in 2 remaining HCC cases (21-22).

FDG PET/CT findings indicated linear hypermetabolism along the portal vein tumor thrombi with an extension of liver tumors in all patients. The mean SUV_max_ of the thrombi was calculated to be 7.0 (range: 4.5-11.5). This result is consistent with the findings of a previous study by Sun L et al., which showed the high metabolism of malignant PVT with the SUV_max_ range of 3.0-11.5 in patients with HCC (21).

In a study by Sharma P et al., a cut-off SUV_max_ of 3.63 was obtained to differentiate tumors from benign venous thrombi with a sensitivity of 72% and a specificity of 90%. Among 5 recorded cases of malignant HCC, complicated by PVT, the portal vein thrombi appeared FDG-avid with the SUV_max_ range of 3.2-9.6. PVT cases were identified to have a linear FDG uptake pattern in 4 out of 5 cases and directly extended from the liver tumors in 3 out of 5 cases (23).

Recently, Hu S et al. investigated the value of FDG PET/CT scan in differentiating between malignant and benign PVT groups in 72 patients with known malignant tumors. They found that SUV_max_ in PVTT (6.37±2.67) was significantly higher than that observed in bland thrombi (2.87±1.47; P<0.01). ROC analysis revealed a cut-off SUV_max_ of 3.35, which could identify malignant PVT cases from the benign type with the sensitivity, specificity, and accuracy of 93.6%, 80.0%, and 88.9%, respectively (24).

Our study demonstrated that FDG-avid PVTT was highly likely to be influenced by direct extension from liver tumors which appeared hypermetabolic, but not significantly higher than PVTT. In a study by Sun L et al., which aimed to assess the ability of FDG PET/CT scan in discrimination between benign and malignant thrombi in HCC patients, the SUVs of malignant thrombi were lower than those of HCC masses; in addition, malignant thrombosis was significantly more common in patients with highly metabolic liver tumor lesions (21).

Enhancement of tumor thrombi and dilatation of the involved portal veins were the principle findings in the dynamic CT images of patients in our study. This result was relatively similar to those reported in previous studies, which demonstrated that the presence of enhancing and expansible thrombosed portal veins in HCC strongly suggests malignant thrombosis (12-13). Malignant thrombi in HCC showed a generalized enhancement in 83% of cases and neovascularity in 43% of cases (12). Shah ZK et al. found the enhancement of PVT in all cases with a final diagnosis of HCC in a study on patients with radiological reports of PVT or portal vein invasion in the presence of hepatic lesions (13).

In the current study, the mean greatest diameter of thrombosed portal veins was 1.8 cm, which was smaller than the result reported by Tublin ME and colleagues. They demonstrated a threshold diameter of ≥23 mm for main portal veins in CT images to discriminate between malignant and benign PVT groups with a sensitivity of 62% and a specificity of 100% in patients with known liver cirrhosis and PVT for preoperative transplant evaluation or potential resection of HCC (12).

In a study by Shah ZK et al., significant expansion of the portal vein, containing a malignant thrombus, was identified, unlike that observed in normal portal veins. The mean maximal diameter on CT and MRI images was 2.2 cm in the thrombosis of right or left portal veins and 2.4 cm in the thrombosis of main portal veins in patients with HCC (13). The above-mentioned difference can be explained by many factors influencing PVTT diameter such as portal hypertension, cirrhosis, complete or partial occlusion of the portal vein, and the patient’s height.

Patients with HCC, complicated by PVTT, had a poor prognosis in our study. Most patients did not receive a specific therapy after PET/CT imaging, and prognostic factors beyond PVTT were not analyzed in the current study. Besides providing diagnostic information, FDG uptake in PVT, induced by HCC, may be a sign of more aggressive tumor behavior.

The median survival time of patients was estimated at 5 months, which was less than that reported by Jia L et al., presenting that the median overall survival of HCC patients with PVTT was 14 months, following PVTT diagnosis; the overall survival was associated with liver function, tumor extension, and treatment of HCC and PVTT (4). On the other hand, in a study by Sun L et al., 4 HCC patients with PVTT died during the follow-up (range:1-3 months) (21).

While some studies have performed dynamic liver contrast-enhanced CT after PET acquisition, we reversed the sequence in the acquisition protocol of PET/CT. Additionally, we changed the acquisition of portal venous phase CT of the liver into that of whole-body CT scan and found some apparent advantages in the modified acquisition protocol.

The contrast-enhanced whole-body CT image sets were helpful in diagnosing and localizing lesions of not only liver, but also other parts of the body; it should be kept in mind that contrast-enhanced CT-based attenuation correction can cause artifacts and quantitative errors, which might affect PET images. In addition, SUV_max_ of the liver tissue was reported to be significantly elevated in PET images, when using contrast-enhanced high-dose CT scan versus unenhanced low-dose CT for attenuation correction (25).

The present study had several limitations. The initial HCC was diagnosed based on histopathologic findings only in some cases. A small number of cases of FDG-avid PVT highly suspecting malignant were enrolled in the study. Biopsy or autopsy for histopathological examination of the portal vein thrombus has not been performed to prove malignant nature, although malignant PVT has been known able to appear without an increased FDG uptake and conversely, benign PVT could also appear an increased FDG uptake (23, 24).

## Conclusion

Contrast-enhanced FDG PET/CT scan, a combination of dynamic contrast-enhanced CT and PET scan in a single examination, was feasible and convenient for the identification of FDG-avid PVTT. The intraluminal filling defect, consistent with the thrombus within the portal vein, expansion of the involved portal vein, contrast enhancement, and linear increased FDG uptake of the thrombus with an extension of liver tumor are the findings of FDG-avid PVTT from HCC. 
